# Carvacrol Effectively Inhibits *Pseudomonas tolaasii* In Vitro and Induces Resistance to Brown Blotch Disease in Postharvest *Agaricus bisporus*

**DOI:** 10.3390/foods13223689

**Published:** 2024-11-19

**Authors:** Lei Zhang, Rui Song, Zixuan Shi, Shuai Yuan, Lu Jiao, Mengsha Ma, Xing Wang, Lin Chen, Xia Liu, Demei Meng

**Affiliations:** 1 Tianjin Key Laboratory of Food Quality and Health, College of Food Science and Engineering, Tianjin University of Science & Technology, Tianjin 300457, China; 22844990@mail.tust.edu.cn (L.Z.); 18331063572@163.com (R.S.); 22845913@mail.tust.edu.cn (Z.S.); yuanshuai@mail.tust.edu.cn (S.Y.); 22844934@mail.tust.edu.cn (L.J.); 23844962@mail.tust.edu.cn (M.M.); ax811@mail.tust.edu.cn (X.W.); liuxia@tust.edu.cn (X.L.); 2 School of Chemistry, Chemical Engineering and Biotechnology, Nanyang Technological University, Singapore 637459, Singapore; chen.lin@ntu.edu.sg

**Keywords:** antibacterial activity, action mechanism, brown blotch disease, carvacrol, disease resistance, edible mushrooms

## Abstract

Carvacrol (CAR), a naturally occurring phenolic monoterpene compound, has recently received attention for its potential use in food preservation. However, whether it is effective in controlling brown blotch disease caused by *Pseudomonas tolaasii* in edible mushrooms is unknown. The results of this study showed that CAR effectively inhibits and kills *P. tolaasii* in vitro by disrupting cell membrane integrity and causing the leakage of cellular components. Intracellular proteins and the DNA of *P. tolaasii* may not be the targets of CAR. CAR fumigation at a concentration as low as 20 μmol L^−1^ CAR effectively inhibited *P. tolaasii*-caused brown blotch disease in *Agaricus bisporus*, accompanied by a decrease in polyphenol oxidase activation, melanin production, and malondialdehyde accumulation. CAR treatment also significantly increased the activities of β-1,4-N-acetyl-glucosaminnidase, three antioxidant enzymes, and phenylpropanoid pathway-related enzymes, as well as promoting the accumulation of phenolic, flavonoid, and lignin substances in mushrooms, thereby inducing the resistance of mushrooms to the disease. These results demonstrate the potential application of carvacrol to control bacterial disease in *A. bisporus* mushrooms.

## 1. Introduction

*Agaricus bisporus* (*A. bisporus*), also known as the white button mushroom, is one of the most widely cultivated mushroom species in the world, and its production accounts for about 1/6 of the total global mushroom production [1,2]. It has a huge market potential and research prospects by virtue of its rich nutritional value and increasing sales volume [3,4,5]. *A. bisporus* is susceptible to infection by *Pseudomonas*, which causes brown blotch disease during cultivation and postharvest transport and storage. Infected mushrooms not only show changes in appearance, flavor, and nutritional value, but their yield and shelf life are also negatively affected. In particular, *Pseudomonas tolasi* (*P. tolaasii*), a common soil bacterium that secretes an extracellular toxin called tolaasin, can cause brown, small, and irregular lesions on mushroom caps that may even coalesce to cover the entire surface of the mushroom [3,6]. Currently, the disease control method in mushrooms mainly focuses on low-temperature and chemical preservation (e.g., sodium hypochlorite, sodium sulfite, and chlorine dioxide), but there are limitations to its practical application in terms of ineffectiveness and safety risks [7]. Hence, there is a need to search for new alternative methods to reduce the development of this disease.

Single or multiple compounds derived from plant essential oils (EOs) are considered promising candidates for reducing the use of chemical agents due to their natural and potential antimicrobial properties [8,9]. Carvacrol (CAR), a naturally occurring phenolic monoterpene compound, is a major active ingredient in the OEs of plants such as thyme (*Thymus vulgaris*) and oregano (*Origanum vulgare*). CAR has been internationally certified for its safety and good antimicrobial activity [10]; it also has the advantages of favorable biocompatibility, a pleasant odor, lipophilic chemicals, and antioxidant properties [11,12,13]. CAR not only exerts inhibitory effects on a wide range of foodborne bacterial pathogens such as *Staphylococcus aureus*, *Escherichia coli*, and *Salmonella* [14,15] but also shows potent activity against many kinds of fungal pathogens, such as *Candida albicans* [16], *Valsa pyri* [17], *Penicillium citrinum* and *Meyerozyma caribbica* [18]. However, previous research on the efficacy of CAR against bacteria as a food preservative, mainly in the systems of meat and meat products [19,20], dairy products [21], and seafood [22], and its effectiveness against *P. tolaasii* in vitro and in harvested horticultural crops, especially postharvest mushrooms, remains unknown. In addition, CAR has great potential for controlling phytopathogenic diseases, such as pear plants [17] and fungal decay in postharvest agricultural products, such as red grapefruit [23], goji fruit [24], wheat [25], and tomato fruit [13]. However, compared to fruits and vegetables, there are relatively few reports on the preservation effect of CAR in edible mushrooms. In particular, edible mushrooms comprise different components to fruits and vegetables, and it is, therefore, uncertain whether the CAR treatment is effective against *P. tolaasii*-caused brown blotch disease in the mushroom context. In addition, the specific mechanism of action of CAR against *P. tolaasii* also has not been elucidated. Several studies indicate that the primary mechanism of CAR in vitro is the disruption of the membrane integrity of bacterial and fungal pathogenic cells, such as *Bacillus cereus* [26], *Listeria monocytogenes* [19], *Aspergillus flavus* [25], and *Colletotrichum fructicola* [27]. Other studies showed that CAR treatment also inhibited respiratory activity in *L. monocytogenes* [19], caused mitochondrial depolarization and DNA damage in *A. flavus* [25], and generated reactive oxygen species (ROS) in *C. fructicola* [27] and *A. flavus* [25]. The present study, therefore, aims to investigate, for the first time, the efficacy of CAR against *P. tolaasii* in vitro and in postharvest *A. bisporus*. In addition, the possible mechanisms of CAR in vitro and in vivo were explored. It is anticipated that the findings of this study will provide a theoretical basis for the further development of postharvest preservation methods for edible mushrooms and the potential application of CAR in the field of edible mushroom preservation.

## 2. Materials and Methods

### 2.1. Bacterial Pathogens

The *P. tolaasii* was inoculated into the beef extract–peptone (NA) medium and incubated at 28 °C for 24 h, activated twice, and set aside. *P. tolaasii* colonies, after activation, were selected and cultured in 100 mL of the NB medium (NA without agar), incubated at 28 °C with shaking at 220 rpm until the logarithmic phase was reached.

### 2.2. In Vitro Antibacterial Activity of CAR Against P. tolaasii

#### 2.2.1. Inhibitory Effect of CAR Treatment on *P. tolaasii* Growth

*P. tolaasii* cultures in the logarithmic growth phase were collected and diluted in an NB medium at a ratio of 1:1000. The prepared bacterial suspensions were treated with 1, 2, 3, 4, 5, 6, and 7 mmol L^−1^ of the CAR aqueous solution, respectively, with sterile water as a control (0 mmol L^−1^ CAR). The mixtures were then inoculated into a 96-well plate and incubated for 12–16 h at 28 °C and 220 rpm with shaking, followed by the measurement of absorbance at 600 nm. Each concentration was tested in triplicate.

#### 2.2.2. Killing Effect of CAR Treatment on *P. tolaasii* Growth

*P. tolaasii* cultures in the logarithmic growth phase were collected and diluted in an NB medium at a ratio of 1:1000. Then, different concentrations of the CAR aqueous solution (0.5, 1, 2, 4, and 8 mmol L^−1^) were used to treat the bacterial suspensions, with sterile water as the control (0 mmol L^−1^ CAR). Subsequently, the prepared bacterial suspensions were added to a 96-well plate and shaken at 28 °C and 220 rpm for 2 h. The resulting bacterial cultures were then plated on an NA medium and incubated while inverted at 28 °C for 12–16 h. Bacterial colony counts were then determined.

#### 2.2.3. Killing Kinetics of CAR Treatment Against *P. tolaasii*

The killing kinetics of CAR against *P. tolaasii* were evaluated by the method improved by Song et al. [28] The collected logarithmic growth phase of *P. tolaasii* was diluted with the NB medium at a ratio of 1:1000 and subsequently treated with 2, 4, and 8 mmol L^−1^ CAR aqueous solutions, with sterile water serving as the control (0 mmol L^−1^ CAR). After thorough mixing, the suspensions were cultured at 28 °C and 220 rpm for 5, 15, 30, 60, and 120 min. Subsequently, equal volumes of the suspensions were plated on NA agar plates and incubated while inverted at 28 °C for 12–16 h before colony counting.

### 2.3. In Vitro Antibacterial Mechanism of CAR Against P. tolaasii

#### 2.3.1. Effect of CAR Treatment on *P. tolaasii* Cell Morphology

Scanning electron microscopy (SEM) was used to observe changes in cell morphology in CAR-treated *P. tolaasii*, following Zhang et al. [29], with minor adaptations. *P. tolaasii* cells in the logarithmic phase were collected, centrifuged to remove the culture medium, resuspended in PBS solutions containing different concentrations of CAR (0, 2, 4, 8 mmol L^−1^), and cultured in a shaking incubator at 28 °C and 220 rpm for 2 h. The bacterial cells were then collected by centrifugation and fixed with 4% glutaraldehyde at 4 °C overnight. After fixation, the samples were dehydrated through a series of ethanol gradients [30%, 50%, 70%, 90%, 100% (*v*/*v*)] for 5 min each and finally resuspended in 100% ethanol. The ethanol-resuspended bacterial cells were dropped onto the SEM sample stubs, air-dried, coated, and observed for cell morphology.

#### 2.3.2. Effect of CAR Treatment on *P. tolaasii* Cell Membrane Integrity

The propidium iodide (PI) uptake assay was performed according to a previously established method [28] to assess the effect of CAR on *P. tolaasii* membrane integrity. CAR (0, 2, 4, 8 mmol L^−1^) was used to treat *P. tolaasii* cells, and harvested cells were resuspended in 50 mmol L^−1^ PBS (pH 7.2), mixed with 5 μmol L^−1^ PI and then incubated for 30 min at 28 °C in the dark. Afterward, the cells were observed and imaged with a fluorescence microscope. PBS-treated cells were used as a negative control and were treated and observed under the same conditions.

#### 2.3.3. Effect of CAR Treatment on Cytoplasmic Leakage of *P. tolaasii*

Cytoplasmic leakage was determined according to a previous method [30] using slight modifications. The bacterial suspensions were treated with different concentrations of CAR (0, 2, 4, 8 mmol L^−1^) at 28 °C and 220 rpm. After treatment for 0, 20, 40, 60, 80, 100, and 120 min, respectively, the supernatants were then collected for nucleic acid, protein, and total sugar measurements. Nucleic acid leakage was assessed by measuring absorbance at 260 nm, protein leakage by the Bradford method [31], and total sugar content by the anthrone–sulfuric acid method [32]. Each sample was used for analysis in triplicate.

#### 2.3.4. Effect of CAR Treatment on the Changes in *P. tolaasii* Cellular Proteins and DNA

As outlined in Section 2.3.1, bacterial suspensions were subjected to varying concentrations of CAR (0, 2, 4, 8 mmol L^−1^) at 28 °C for 2 h with agitation at 220 rpm, after which they were rinsed with physiological saline solution. SDS-PAGE and Coomassie Brilliant Blue staining were then conducted, and imaging was performed using the Champ Gel 5000 system (Surwit Technology Co., Ltd., Hangzhou, China) for visualization.

The DNA extraction of *P. tolaasii* cells was conducted using the DNA Extraction Kit (Solarbio, Beijing, China). The extracted DNA was then treated with varying concentrations of CAR (0, 2, 4, 8 mmol L^−1^) in a 1:1 ratio, followed by a three-hour incubation at 28 °C. The samples were finally subjected to 0.8% (*w*/*v*) agarose gel electrophoresis, which was then imaged for observation.

### 2.4. Effect of Postharvest CAR Treatment on the Induction of Resistance to Brown Blotch Disease in A. bisporus Mushrooms

#### 2.4.1. Treatment of *A. bisporus* Mushrooms

Fresh *A. bisporus* mushrooms were procured from Tianshui Zhongxing Bio-Technology Co., Ltd. (Lanzhou, Gansu, China). Following pre-cooling at 4 °C, 600 mushrooms of uniform size, devoid of cap opening, browning, mechanical damage, and disease, were selected. The mushroom stipes were cut evenly to a uniform length and washed with distilled water before air-drying. Subsequently, *A. bisporus* was inoculated with *P. tolaasii*, as previously described [33], and the mushrooms were randomly divided into three groups. According to our preliminary tests, CAR treatments with a concentration of ≥30 μmol L^−1^ could cause the white mushroom to turn yellow, so CAR with concentrations of 10 and 20 μmol L^−1^ was selected for mushroom treatments. Specifically, the three groups of mushrooms were covered with a preservation paper wrap that had been soaked with 0 (control), 10, and 20 μmol L^−1^ of CAR solution, respectively, and packaged in a plastic film. The three treatment groups of *A. bisporus* (each with three replicates) were stored under conditions of 85–95% relative humidity and 4 °C temperature. At 0, 12, 24, 36, 48, 60, 72, and 84 h, the number and index of mushrooms developing disease were recorded to calculate the incidence rate and disease index according to the previous method [33], and seven mushrooms were randomly selected and sampled for subsequent analysis in the experiment. The cap lesion tissue, approximately 2 mm thick, was excised and chopped into small pieces to determine the melanin content; the adjacent healthy tissue, approximately 7 mm thick, was immediately exercised and prepared for the determination of the other indicators.

#### 2.4.2. Measurement of MDA, Melanin, Lignin, Total Phenolic, and Flavonoid Content

The MDA content of the mushrooms was determined using the thiobarbituric acid (TBA) method [34]. One gram of the mushroom samples was ground with 5 mL of trichloroacetic acid (10% *v*/*v*), centrifuged at 4 °C and 13,000× *g* for 15 min, and the supernatants were mixed with trichloroacetic acid containing 0.5% (*w*/*v*) TBA (10% *v*/*v*). Subsequently, the mixture was incubated in a boiling water bath for 20 min, followed by centrifugation to collect the supernatant. Absorbance was measured at wavelengths of 532 nm, 600 nm, and 450 nm to calculate the MDA content.

The melanin content of the mushrooms was analyzed following a method previously described by Selvakumar et al. [35] and Sun et al. [36] with some modifications. One gram of mushroom samples was thoroughly ground with 3 mL of 1 mol L^−1^ NaOH, then subjected to high-pressure sterilization at 121 °C for 20 min. Subsequently, the mixture was centrifuged at 8000× *g* for 5 min to collect the supernatants, which were subsequently adjusted to a pH of 2.0 by the addition of 7 mol L^−1^ HCl. Following centrifugation at 8000× *g* for 5 min and the removal of the supernatants, the precipitates were collected and then mixed with 7 mol L^−1^ HCl and placed in a boiling water bath for 20 min. Following centrifugation at 8000× *g* for 5 min, the melanin precipitates were washed three times with sterile water, air dried, and finally dissolved in 1 mol L^−1^ NaOH for the absorbance measurement at 400 nm. Each sample was analyzed in triplicate. A standard curve for melanin was concurrently plotted to calculate the melanin content in the samples based on the equation of the standard curve.

The lignin content of the mushrooms was determined by means of a previously established method [37]. One gram of the sample was ground in 2.0 mL of 95% ethanol solution and centrifuged at 8000× *g* for 7 min at 4 °C. Subsequently, the supernatants were discarded, and the resulting precipitate was washed three times with 95% ethanol and ethanol–hexane (1:2, *v*/*v*), respectively. It was then redissolved and incubated in a water bath at 70 °C for 30 min in 0.5 mL of 25% (*v*/*v*) bromoacetyl solution. We then added 0.9 mL of 2 mol L^−1^ NaOH to terminate the reaction. Subsequently, 5 mL of glacial acetic acid and 0.1 mL of 7.5 mol L^−1^ hydroxylamine hydrochloride were added, and the reaction was centrifuged at 8000× *g* for 7 min. Finally, the absorbance of the supernatant was measured at 280 nm. The lignin content in each sample was calculated from the standard curve of the lignin standard and expressed as grams per kilogram of fresh weight (g kg^−1^ FW). The assay was repeated three times for each sample.

The total phenolic and flavonoid content of the mushrooms was determined in accordance with the methodology described in Yang et al. [33], with certain modifications. The sample tissues were ground with a 1% (*v*/*v*) hydrochloric acid–methanol solution in an ice bath and incubated at 4 °C for 2 h in the dark. Subsequently, the supernatants were centrifuged at 8000× *g* for 15 min at 4 °C. Subsequently, absorbance measurements at 280 nm and 325 nm were carried out, and standard curves for gallic acid and catechin were used to calculate the total phenolic and flavonoid contents (g kg^−1^ FW), respectively.

#### 2.4.3. Enzyme Activity Assays

Peroxidase (POD), superoxide dismutase (SOD), polyphenol oxidase (PPO), and catalase (CAT) were measured using assay kits A084-3-1, A001-1-1, A136-1-1, and A007-1-1, respectively, which were purchased from Nanjing Jiancheng Bioengineering Institute (Nanjing, Jiangsu, China).

To extract β-1,4-N-acetyl-glucosaminnidase (NAG), 1.0 g of the mushroom samples was thoroughly ground in 3 mL of a 100 mmol L^−1^ acetic acid–sodium acetate buffer (pH 5.0) at 4 °C and centrifuged at 8000× *g* for 15 min. Subsequently, the supernatants were mixed with a 100 mmol L^−1^ acetic acid-sodium acetate buffer (pH 5.0, containing 300 mg L^−1^ of p-nitrophenyl-N-acetyl-β-D-glucosaminide). After incubation for 30 min at 37 °C, 0.4 mol L^−1^ Na_2_CO_3_ was added to terminate the reaction, and the absorbance of the mixture was measured at 405 nm. Each sample was measured three times. The amount of p-nitrophenol generated was measured from the standard curve, and NAG activity was defined as micromoles per hour per milligram of protein (μmol h^−1^ mg^−1^ protein).

The extraction of phenylalanine ammonia-lyase (PAL) was conducted using a 200 mmol L^−1^ borate buffer (pH 8.8), and the activity was determined according to the method of Ji et al. [38] One unit (U) of PAL activity represents a change in absorbance at 290 nm of 1.0 per hour.

The extraction of cinnamic 4-coumarate-CoA ligase (4CL) and acid-4-hydroxylase (C4H) was conducted using a 50 mmol L^−1^ Tris-HCl buffer (pH 8.9). Subsequently, the 4CL and C4H activity assays were performed in accordance with the methodologies described by Ji et al. [38] and Wang et al. [39], respectively. One unit (U) of C4H activity represents a 0.001 change in absorbance at 340 nm per hour, while one unit (U) of 4CL activity represents a 0.01 change in absorbance at 333 nm per hour.

The extraction of cinnamyl alcohol dehydrogenase (CAD) was conducted using a 0.2 mmol L^−1^ Tris-HCl buffer (pH 6.25), and the activity was determined according to the method of Li et al. [40]. One unit (U) of CAD activity represents 0.01 of change in absorbance at 340 nm per hour.

### 2.5. Statistical Analysis

The experimental data were subjected to statistical analysis using analysis of variance (ANOVA) with IBM SPSS Statistics version 25.0.

## 3. Results

### 3.1. CAR Treatment Showed a Strong Inhibitory and Killing Effect on the Growth of P. tolaasii In Vitro

**Figure 1:**
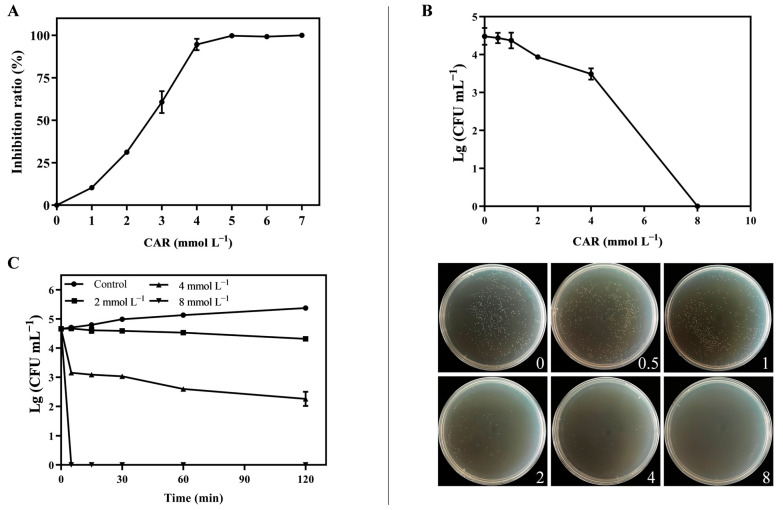
Antibacterial activity of CAR against *P. tolaasii* in vitro. (**A**) The inhibition ratio of different concentrations of CAR on *P. tolaasii* growth. (**B**) Killing effect of different concentrations of CAR against *P. tolaasii*. (**C**) Killing kinetics of CAR against *P. tolaasii*. Data represent the means ± SD, *n* = 3.

As shown in Figure 1A, CAR treatment significantly inhibited the growth of *P. tolaasii*, and increasing concentrations of CAR resulted in an increasing inhibitory effect. When the concentration of CAR reached 4 and 5 mmol L^−1^, the inhibition rate exceeded 90% and 99%, respectively. Figure 1B shows the killing effect of different concentrations of CAR on *P. tolaasii*. When the concentration of CAR increased to 8 mmol L^−1^, no bacterial colonies were observed on the plate, indicating that 100% of *P. tolaasii* was effectively killed. To further verify the killing efficiency of CAR on *P. tolaasii*, the killing kinetic assays were performed (Figure 1C). The control group showed a continuous increase in colony count over the incubation period, while the CAR-treated groups showed a decreasing trend. In particular, 4 and 8 mmol L^−1^ CAR showed a rapid killing effect during the first 5 min, and 8 mmol L^−1^ CAR was able to kill all *P. tolaasii* within 5 min. These results indicate that CAR has a potent inhibitory and killing effect against *P. tolaasii* in vitro.

### 3.2. CAR Treatment Altered the Cellular Morphology of P. tolaasii

**Figure 2:**
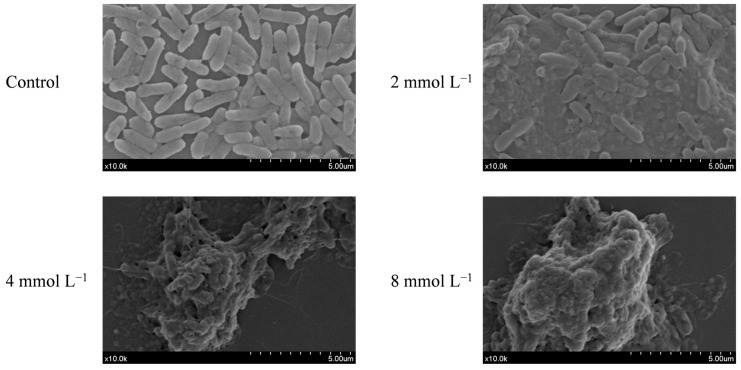
Effect of CAR treatment on the cell morphology of *P. tolaasii* in the 0 (control), 2, 4 or 8 mmol L^−1^ of CAR treatment groups.

The SEM observation of the cellular morphology of *P. tolaasii* revealed that different concentrations of CAR caused different degrees of damage to the cellular morphology of *P. tolaasii* compared to the control group (Figure 2). Untreated *P. tolaasii* cells appeared plump with a smooth surface. Treatment with 2 mmol L^−1^ CAR resulted in some *P. tolaasii* cells losing their basal shape and exhibiting fragmentation, adhesion, and aggregation. As the concentration of CAR increased, the degree of cell fragmentation also increased. When the treatment concentration reached 8 mmol L^−1^, *P. tolaasii* completely lost its cellular morphology, and the cells were completely fragmented and adherent. These results indicate the destructive effect of CAR on *P. tolaasii* cells.

### 3.3. CAR Treatment Disrupts the Membrane Integrity of P. tolaasii

**Figure 3:**
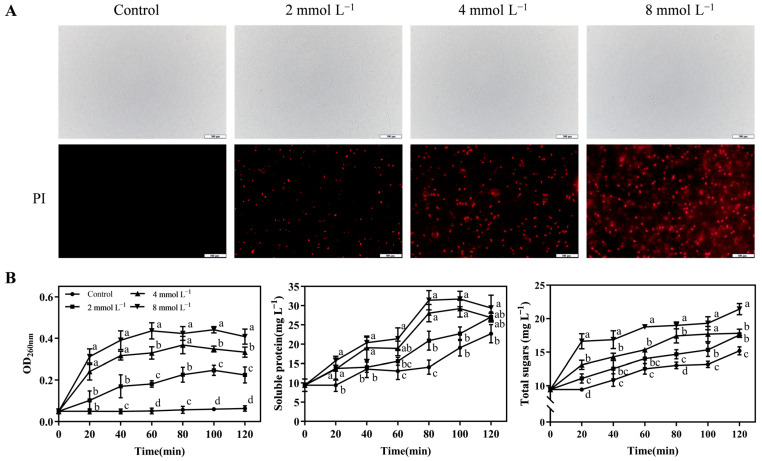
Effect of CAR treatment on the plasma membrane of *P. tolaasii* cells. (**A**) Fluorescent microscope observations of PI uptake into *P. tolaasii* cells in 0 (control), 2, 4 or 8 mmol L^−1^ of the CAR treatment groups. (**B**) The leakage of cytoplasmic leakage of nucleic acid (absorbance at 260 nm), soluble proteins, and total sugars from *P. tolaasii* in 0 (control) at 2, 4, or 8 mmol L^−1^ of CAR treatment groups during the 120 min duration. Data represent the means ± SD, *n* = 3. Different lowercase letters indicate groups with significant differences at *p* < 0.05.

Based on the SEM observation results, *P. tolaasii* was further subjected to PI staining and fluorescence signal detection. PI cannot penetrate intact cell membranes for staining, but it can pass through damaged membranes, bind to the DNA inside the cell, and show red fluorescence. As shown in Figure 3A, *P. tolaasii* treated with PBS showed no red fluorescence signal, while the three groups treated with different concentrations of CAR showed an obvious red fluorescence signal. In particular, 8 mmol L^−1^ CAR showed the strongest signal compared to the bacterial cells treated with 2 mmol L^−1^ and 4 mmol L^−1^ CAR. These results suggest that CAR treatment can disrupt the integrity of *P. tolaasii* bacterial cell membranes, and the degree of disruption increases with higher concentrations of CAR treatment. We also evaluated cell membrane damage by measuring the leakage of nucleic acids, proteins, and soluble carbohydrates within the *P. tolaasii* cells (Figure 3B). Higher absorbance values at 260 nm and higher levels of soluble protein and total sugars were consistently detected in the supernatants of cells treated with 2, 4, and 8 mmol L^−1^ CAR compared to cells without CAR treatment (control) at all time points over a 120 min period. In addition, OD260, a soluble protein, and total sugar levels were increased by CAR treatment in a concentration-dependent manner, with 8 mmol L^−1^ CAR resulting in the highest increase. Taken together, our results demonstrate that CAR treatments can disrupt the integrity of *P. tolaasii* cell membranes, resulting in the leakage of cytoplasmic constituents.

### 3.4. CAR Treatment Did Not Affect the Intracellular Proteins and DNA of P. tolaasii

**Figure 4:**
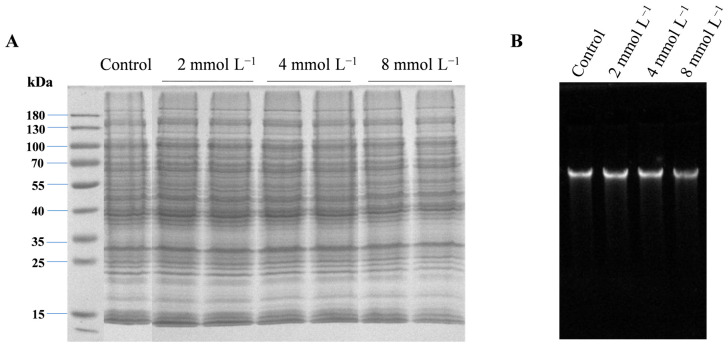
Changes in intracellular proteins (**A**) and DNA (**B**) of *P. tolaasii* cells after 0 (control), 2, 4, or 8 mmol L^−1^ of CAR treatment by SDS-PAGE analysis and the DNA binding assay.

To investigate whether CAR acts against pathogens by interacting with the intracellular proteins or DNA of *P. tolaasii*, we next performed SDS-PAGE analysis and DNA binding assays. As shown in Figure 4A, there were no clear changes in the protein bands after different concentrations of CAR treatments, indicating that CAR treatments did not affect the synthesis and stability of intracellular proteins. Similarly, the gel retardation assays showed that all the CAR treatments also had no significant effect on the *P. tolaasii* DNA migration (Figure 4B), indicating that CAR could not interact with or degrade *P. tolaasii* DNA.

### 3.5. CAR Treatment Inhibited P. tolaasii-Caused Brown Blotch Disease in A. bisporus Mushrooms

**Figure 5:**
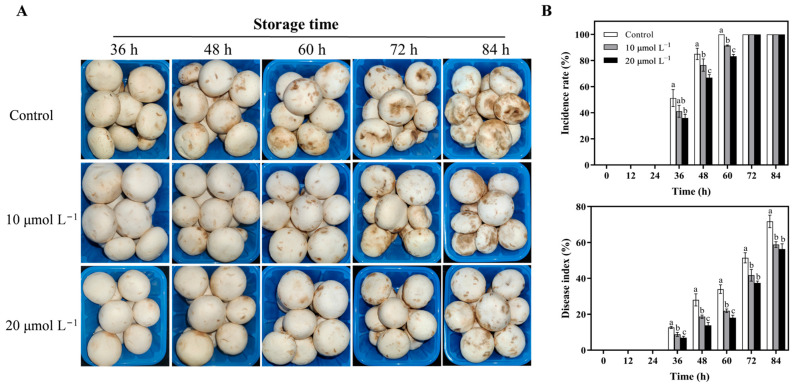
Postharvest treatment with CAR suppressed brown spot disease in mushrooms caused by *P. tolaasii*. The figure shows the visual symptoms (**A**), incidence rate, and disease index (**B**) of mushrooms treated with 0 (control), 10, and 20 μmol L^−1^ CAR during storage. Data represent the means ± SD, *n* = 3. Different lowercase letters indicate groups with significant differences at *p* < 0.05.

To determine the effect of CAR treatments on the development of bacterial brown blotch disease in edible mushrooms, the sporophores of *A. bisporus* were treated with CAR. The control group showed disease spots 36 h after inoculation, and the spots became progressively larger and darker as the storage time increased (Figure 5). In contrast, the mushrooms treated with 10 and 20 μmol L^−1^ CAR began to show clear symptoms of the disease at 48 h post-inoculation, with the severity of the disease being significantly less in the 20 μmol L^−1^ CAR treatment group than in the 10 μmol L^−1^ treatment group (Figure 5A). Furthermore, both concentrations were found to reduce the incidence and index of disease during storage. Notably, the 20 μmol L^−1^ CAR-treated group showed the lowest disease incidence and index and achieved the most effective disease control (Figure 5B). The incidence rate of the control group mushrooms reached a 100% incidence rate at 60 h, while the 10 μmol L^−1^ and 20 μmol L^−1^ treatment groups were only 91.4 % and 82.5%, respectively. After 60 h of storage, the disease index in the control group was approximately 1.55 times and 1.88 times higher than that in the 10 μmol L^−1^ and 20 μmol L^−1^ groups, respectively. After 72 h of storage, the disease index in the control group exceeded 50%, while the 20 μmol L^−1^ CAR-treated group showed only 37.4%. Collectively, CAR treatment prevented the development of brown blotch disease in *A. bisporus* mushrooms caused by *P. tolaasii*.

### 3.6. CAR Treatment Inhibited P. tolaasii-Caused PPO Activation and Melanin Production and Reduced MDA Accumulation in A. bisporus Mushrooms

**Figure 6:**
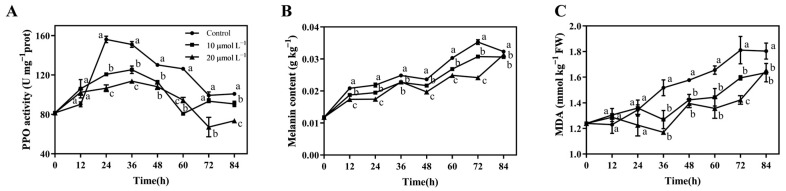
Effects of postharvest CAR treatments on PPO activity (**A**), melanin content (**B**), and MDA content (**C**) in *A. bisporus* after inoculation with *P. tolaasii*. Data represent the means ± SD, *n* = 3. Different lowercase letters indicate groups with significant differences at *p* < 0.05.

The PPO catalyzes the oxidation of phenolic compounds in living organisms to quinones, resulting in browning [41,42]. Tolaasin secreted by *P. tolaasii* can disrupt the integrity of cell membranes and activate PPO, resulting in the appearance of brown lesions on the surface of infected mushrooms [43,44]. Throughout the storage period, the trend of PPO activity showed an initial increase followed by a decrease, as shown in Figure 6A. In the control group, PPO activity increased sharply after 12 h and peaked at 24 h. However, both the 10 μmol L^−1^ and 20 μmol L^−1^ CAR treatment groups delayed the peak PPO activity to 36 h. It is noteworthy that the peak value in the 10 μmol L^−1^ and 20 μmol L^−1^ CAR treatment groups was only 80% and 72% of that in the control group, respectively. In addition, the PPO activity in the 20 μmol L^−1^ CAR treatment group was the lowest at almost all the time points.

Melanin content is a product of the browning reaction that can visually reflect the degree of disease development in *A. bisporus*. Figure 6B shows that with a prolonged storage time, the melanin content gradually increased in all three treatment groups. Consistent with PPO activity, the melanin content in the 10 μmol L^−1^ and 20 μmol L^−1^ CAR treatment groups was also consistently lower than the control group throughout the storage, with the lowest values in the 20 μmol L^−1^ CAR treatment group. The above results indicate that CAR treatment could inhibit the *P. tolaasii*-induced PPO activation and, hence, melanin production in *A. bisporus* mushrooms.

In addition to melanin, the MDA content also showed an overall increasing trend during the storage process, reaching its peak at 84 h, indicating the continuous accumulation of MDA in *A. bisporus* mushrooms. In the control group, the MDA content exhibited a marked increase from 12 h onwards, reaching levels 1.28 and 1.47 times higher than the initial value after 48 and 72 h of storage, respectively. In contrast, only a very slight increase in MDA content was detected in the CAR treatment groups during the first 48 h of storage. Therefore, CAR treatment could also inhibit the MDA accumulation in *A. bisporus* mushrooms.

### 3.7. CAR Treatment Increased CAT, POD, SOD, and NAG Activity in Postharvest A. bisporus to Resist P. tolaasii Infection

**Figure 7:**
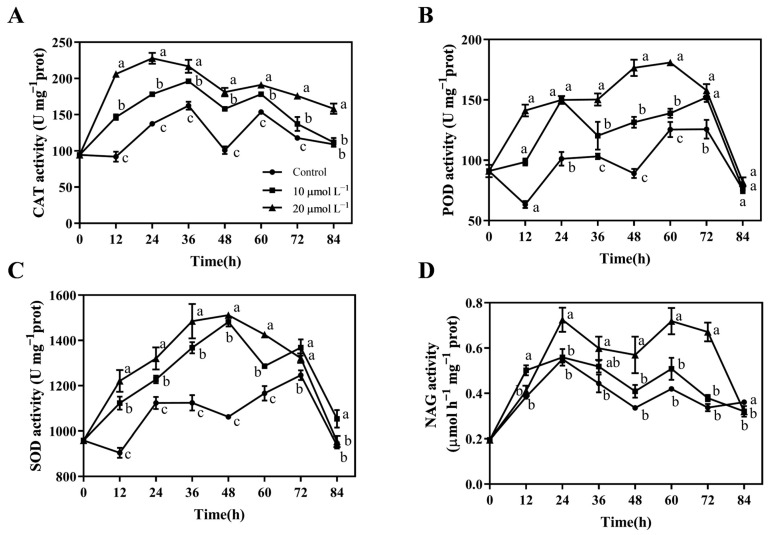
Effects of postharvest CAR treatments on the activities of CAT (**A**), POD (**B**), SOD (**C**), and NAG activity (**D**) in *A. bisporus* after inoculation with *P. tolaasii*. Data represent the means ± SD, *n* = 3. Different lowercase letters indicate groups with significant differences at *p* < 0.05.

Our previous research has shown that the enhancement of antioxidant enzyme activities is beneficial for the postharvest resistance of mushrooms to bacterial brown blotch disease [33]. Hence, to investigate whether CAR could induce the disease resistance of *A. bisporus* mushrooms against bacterial brown blotch disease, we performed the measurements and analysis of antioxidant enzyme activities, including CAT, POD, and SOD. Figure 7A–C show that within 84 h of storage, the CAR-treated groups had consistently higher activities of CAT, POD, and SOD than the control group, with the 20 μmol L^−1^ CAR group being higher than the 10 μmol L^−1^ CAR group. From 12 to 48 h after inoculation, the CAT activity in the 20 μmol L^−1^ CAR treatment group was found to be approximately 1.33 to 2.24 times higher than that in the control group. Similarly, the POD and SOD activities in the 20 μmol L^−1^ CAR treatment group were 1.46 to 2.23 and 1.18 to 1.42 times higher, respectively, than those in the control group at 12 to 60 h post-inoculation.

Since *A. bisporus* could secrete NAG, which is an extracellular enzyme capable of degrading bacteria, we next investigated whether CAR treatment could induce NAG to degrade *P. tolaasii*. Figure 7D shows that NAG activity remained higher in the CAR-treated groups than in the control group from 24 h to 72 h during storage. At 60 h, the NAG enzyme activity in the 10 and 20 μmol L^−1^ CAR-treated groups was 1.21 and 1.71 times higher than that in the control group, respectively. The aforementioned results indicated that CAR treatment was effective in inducing both antioxidant enzymes and the bacterial degrading enzyme for defense against *P. tolaasii* infection.

### 3.8. CAR Treatment Promoted the Phenylpropanoid Pathway in Postharvest A. bisporus to Resist P. tolaasii Infection

**Figure 8:**
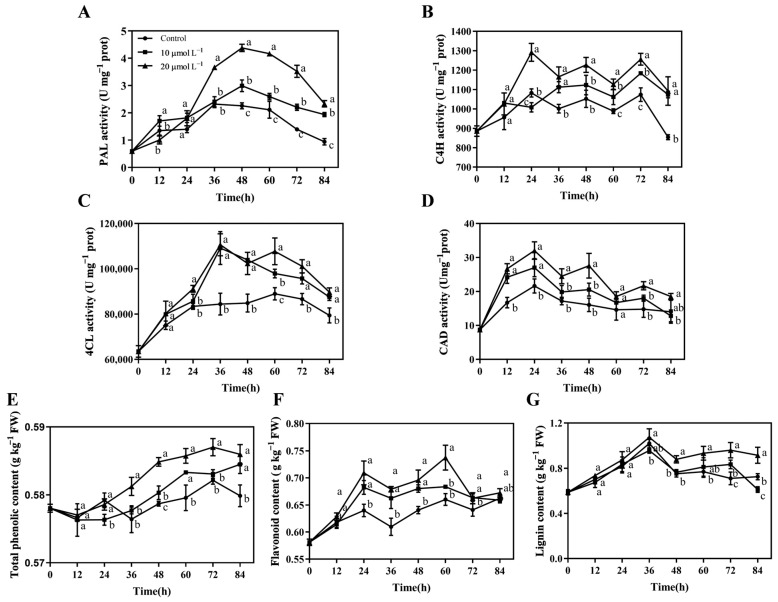
Effects of postharvest CAR treatments on the activities of PAL (**A**), C4H (**B**), 4CL (**C**), and CAD (**D**), and the contents of total phenolics (**E**), flavonoids (**F**), and lignin (**G**) in *A. bisporus* after inoculation with *P. tolaasii*. Data represent the means ± SD, *n* = 3. Different lowercase letters indicate groups with significant differences at *p* < 0.05.

Secondary metabolites (e.g., phenolics and flavonoids) produced in the phenylpropanoid pathway play a pivotal role in plant responses to both biotic and abiotic stressors [45]. Therefore, the effect of CAR treatment on the phenylpropanoid pathway in postharvest *A. bisporus* mushrooms was investigated in the current study. As illustrated in Figure 8, the activities of key enzymes (including PAL, C4H, 4CL, and CAD) in the phenylpropanoid pathway were all markedly elevated by CAR treatments during the 84 h storage period in comparison to the control group, resulting in a notable increase in the levels of total phenolics, flavonoids, and lignin. Furthermore, with respect to all indicators depicted in Figure 8, CAR at 20 μmol L^−1^ exhibited a more pronounced promoting effect than the 10 μmol L^−1^ CAR treatment on the phenylpropanoid pathway.

Specifically, CAR treatments significantly promoted the increase in PAL activity during the second half of the storage period (36–84 h), with the peak value in the 20 μmol L^−1^ CAR-treated groups being 1.95 times higher than that of the control group (Figure 8A). CAR treatments led to a significant increase in C4H activity from 24 h onwards, with the activity in the 20 μmol L^−1^ CAR-treated group being 113% to 128% of that in the control group (Figure 8B); 4CL activity in the 10 μmol L^−1^ and 20 μmol L^−1^ CAR treatments were approximately 1.03–1.31 times higher than that of the control from 24 h to 84 h (Figure 8C). CAD activity was rapidly elevated by CAR treatments during the first half of storage (12–48 h), and the activity in the 20 μmol L^−1^ CAR-treated group at 12 and 24 h was approximately 1.60 and 1.48 times higher than that of the control, respectively (Figure 8D).

It was observed that total phenolics and flavonoids were produced and accumulated during the defense process of *A. bisporus* against *P. tolaasii* (Figure 8E,F). Following inoculation, the total phenolic content in the 20 μmol L^−1^ CAR-treated group was approximately 1.01 times higher than that of the control group over the 36–84 h that followed. Similarly, the total flavonoid content in the 20 μmol L^−1^ CAR-treated group was approximately 1.09–1.12 times higher than that of the control group during the 24 h to 60 h post-inoculation period (Figure 8E,F). In comparison to the control group, the lignin content of the 10 μmol L^−1^ CAR-treated group exhibited a significant increase only at 72 h of storage (Figure 8G). In contrast, the 20 μmol L^−1^ CAR-treated group demonstrated a significantly higher lignin content than the control group from 48 h onwards, with the greatest increase observed being 135% of the control at 72 h. It can be concluded that the CAR treatment activated the phenylpropanoid pathway, which may contribute to the resistance of *A. bisporus* to infection by *P. tolaasii*. This was evidenced by the elevated activities of associated enzymes and the accumulation of metabolic substances.

## 4. Discussion

Brown blotch disease, caused by the bacterial pathogen *P. tolaasii*, is a key factor leading to the postharvest deterioration of edible mushrooms, and currently, brown blotch disease has been identified in various edible mushrooms [46]. CAR, as a promising environmentally friendly antimicrobial substance, has recently received widespread attention for its potential use in food preservation [47]. However, to date, the antimicrobial activity of CAR against the pathogen *P. tolaasii* in vitro and in edible mushrooms has not been investigated.

The present study first demonstrated that CAR not only has a strong inhibitory effect on the growth of *P. tolaasii* but also can completely kill the pathogens within 5 min when 8 mmol L^−1^ of CAR is used (Figure 1). This result is consistent with previous reports, which documented that the minimum inhibitory concentration (MIC) and minimum bactericidal/fungicidal concentration (MBC/MFC) of CAR against bacterial and fungal pathogens range from 78 to 1250 mg L^−1^ [12,48,49]. Notably, subsequent observations revealed that the concentration of CAR required to inhibit the brown blotch disease of *A. bisporus* caused by *P. tolaasii* was considerably lower than that required in the in vitro experiments. A 20 μmol L^−1^ CAR treatment was observed to significantly inhibit the development and expansion of the brown spots on the cap of the mushrooms, as well as reduce the incidence rate and disease index (Figure 5). Treatments with a concentration of ≥ 30 μmol L^−1^ were observed to result in the white mushroom turning yellow and exacerbating the disease. Previous researchers have also highlighted the potential for CAR with a high concentration to have adverse effects on fruits and phytotoxic effects on crop plants due to its characteristics of small particle size and effective penetration capability [23,50]. Therefore, the utilization of an appropriate, yet not excessive, concentration of CAR is advised for the control of mushroom diseases in practical applications.

Given the inherently volatile nature of CAR and the necessity to minimize the adverse effects of CAR on mushroom tissues, a fumigation method was employed in the current study for the treatment of mushrooms with CAR. The inhibitory effects of CAR on *Aspergillus flavus* infection during wheat storage were also previously investigated by Duan et al. [25] using a fumigation method. Following CAR fumigation, a significant reduction in disease incidence and index was observed, particularly in the period preceding 60 h post-inoculation. In the 20 μmol L^−1^ CAR-treated mushrooms, the disease index remained consistently below 55% of the control value (Figure 5). At 72 and 84 h, the disease index reached a value of 72.8 and 78.6% in the control group, respectively, indicating a gradual decline in the efficacy of CAR in inhibiting mushroom disease as time progressed. To enhance the stability and residence time of CAR, several researchers have recently explored the incorporation of CAR onto specific carriers [13,51,52], the encapsulation of CAR using stabilizers [53], and the development of active packaging utilizing CAR as a component [54]. For example, Sánchez-Hernández et al. [13] discovered that the chitosan-carboxymethylcellulose-alginate nanocarriers encapsulated with CAR required lower concentrations of CAR to achieve the same inhibitory effect. The MIC values for the encapsulated CAR treatments were 23.3 to 31.3 µg mL^−1^ against *Botrytis cinerea*, *Penicillium expansum*, and *Colletotrichum coccodes*, whereas the unencapsulated CAR treatments exhibited MIC values of 500 to 1500 µg mL^−1^. Consequently, a practical dose of CAR (50–100 µg mL^−1^) was found to provide complete protection in postharvest tomatoes against pathogens, effectively resolving the controversy surrounding the application of high doses of CAR in crop protection. Similarly, Fang et al. [54] reported that the combination of CAR with a whey protein isolate emulsion and ε-polylysine (ε-PL) had a beneficial effect on the storage stability of CAR. The prepared CAR/ε-PL-loaded nanoemulsion was effective in suppressing the formation of *Staphylococcus aureus* and *Escherichia coli* biofilms and in preserving mangoes from black rot during a seven-day storage period. Therefore, further research is required to explore different forms or combinations of CAR utilization, such as encapsulating and combination methods, to achieve a more effective control effect in the preservation of edible mushrooms.

It has been shown previously that CAR can exert antibacterial effects by altering the permeability of bacterial membranes [12]. Therefore, in the current study, the effects of CAR on the cytoplasmic membrane of *P. tolaasii* were also investigated. As shown in Figure 3, CAR treatments induced the disruption of *P. tolaasii* membranes accompanied by the leakage of vital intercellular components, including nucleic acids, soluble proteins, and soluble carbohydrates, consequently resulting in significant damage to the cellular morphology of *P. tolaasii* (Figure 2). Furthermore, the degree of cell disruption and leakage was in a concentration-dependent manner after CAR treatment. Previous reports also indicated that CAR exhibited similar bactericidal mechanisms against *Pseudomonas aeruginosa* [48] and *Dickeya zeae* [55]. Therefore, it could be inferred that CAR inhibits the growth of *P. tolaasii* by disrupting the plasma membranes and inducing the release of intracellular components. To further investigate whether CAR can target cellular components, we examined the effects of CAR on intracellular proteins and DNA in *P. tolaasii* cells. It was found that CAR treatment did not alter the bands of intracellular proteins and DNA (Figure 4). Yin et al. [56] found that CAR disrupted *Lasiodiplodia theobromae*’s plasma membrane and bound to its DNA, thereby controlling tea leaf disease. This differs from our findings, likely due to the differences in the characteristics of the pathogen itself. In addition, CAR also has other antimicrobial mechanisms, such as inducing endoplasmic reticulum stress [57] and producing reactive oxygen species [23]. Therefore, further in-depth investigations are needed in the future to determine whether CAR can kill *P. tolaasii* by other mechanisms.

It is of particular significance that the present study has demonstrated that the application of CAR has the potential to enhance disease resistance against brown blotch disease in postharvest *A. bisporus* mushrooms. This is achieved by increasing the activities of three antioxidant-related enzymes (CAT, POD, and SOD) and one bacterial degradation-related enzyme (NAG), as well as promoting pathway-related enzyme activities (PAL, C4H, 4CL, and CAD) and the accumulation of phenolic, flavonoid, and lignin substances (Figure 7 and Figure 8). Consequently, CAR treatments effectively inhibited *P. tolaasii*-induced PPO activation and melanin production, as well as MDA accumulation in *A. bisporus* mushrooms (Figure 6). The phenylpropanoid pathway exerts important roles in the defense system and postharvest quality improvement of mushrooms [58,59,60]. The secondary metabolites of the phenylpropanoid pathway, total phenolics, and flavonoids, are important active substances that exert antibacterial and antioxidant effects [61]. In our previous study, the activities of CAT, POD, and SOD, as well as the phenylpropanoid pathway, were also shown to play an important role in the defense response of *A. bisporus* against *P. tolaasii* [33] and ε-PL-induced resistance [28]. In agreement with our findings, a similar phenomenon was reported in postharvest kiwifruit, where CAR treatment significantly increased CAT, POD, SOD, PAL, C4H, and 4CL activities and increased total phenolic and flavonoid contents, thereby inducing fruit resistance to stem-end rot [62]. Similarly, Li et al. [23] reported that CAR treatment enhanced the resistance of red grapes to the decay disease caused by *Alternaria alternata* during storage by increasing the activities of antioxidant response-related enzymes (CAT, POD, and SOD) and a PAL enzyme. It is well known that chitinase and glucanase are hallmark enzymes that are involved in plant resistance to fungal pathogens due to their ability to directly hydrolyze the fungal cell wall [63,64]. However, there is a paucity of information regarding the pathogenesis-related enzymes associated with resistance to bacterial pathogens. It is worth mentioning that NAG was observed to be significantly induced by CAR treatment against *P. tolaasii* pathogens, indicating, for the first time, that it is an important bacterial defense-related enzyme in edible mushrooms, with the ability to directly degrade bacterial pathogens. Nevertheless, further study is required to determine whether there are other pathogenesis-related proteins involved.

## 5. Conclusions

The present study demonstrates the potent antibacterial activity of CAR against *P. tolaasii* in vitro and its efficacy in controlling the brown blotch disease of postharvest *A. bisporus* mushrooms caused by *P. tolaasii.* The primary mechanism of action of CAR against *P. tolaasii* is the disruption of cell membranes. Moreover, the current results indicate that exogenous CAR treatment can induce resistance to bacterial brown blotch disease by increasing the activities of antioxidant-related enzymes, a bacterial degradation-related enzyme, and phenylpropanoid pathway-related enzymes, as well as by promoting the accumulation of phenolic, flavonoid, and lignin substances in the mushrooms. Therefore, CAR has promising potential for the control of bacterial brown blotch disease in edible mushrooms. In subsequent studies, we aim to investigate strategies for optimizing the efficacy of CAR in the control of mushroom diseases.

## Data Availability

The original contributions presented in the study are included in the article; further inquiries can be directed to the corresponding author.
